# Neuropsychological disposition and its impact on the executive functions and cognitive style in patients with obsessive-compulsive disorder

**DOI:** 10.4103/0019-5545.31598

**Published:** 2006

**Authors:** Sreemoyee Tarafder, Pallabi Bhattacharya, Debika Paul, Gautam Bandyopadhyay, Pritha Mukhopadhyay

**Affiliations:** * Research Scholar Department of Psychology, University of Calcutta; ** Research Scholar Department of Psychology, University of Calcutta; *** Research Scholar Department of Psychology, University of Calcutta; **** Assistant Professor Department of Psychiatry, R.G. Kar Medical College and Hospital, Kolkata; ***** Reader Department of Psychology, University of Calcutta

**Keywords:** Neuropsychology, executive function, cognitive style, obsessive–compulsive disorder, subcortical–cerebeller–spinal domain

## Abstract

**Background:**

Recent brain imaging and electrophysiological studies have consistently shown dysfunction of the fronto-striatal thalamic pathways in patients with obsessive–compulsive disorder (OCD).

**Aim:**

To study the relationship of neuropsychological disposition with the executive functions and cognitive style in patients with OCD.

**Methods:**

Twenty OCD patients (14 males, 6 females) and 20 normal control subjects, matched for all relevant variables including age, sex and education, were studied. Neuropsychological disposition was assessed on the Adult Neuropsychological Questionnaire (ANQ), the executive functions were assessed through Wisconsin Card Sorting Test (WCST), and the cognitive style was assessed by employing the Embedded Figure Test (EFT).

**Results:**

Subcortical–cerebellar–spinal domain of ANQ was found to be associated with cognitive style and executive functions.

**Conclusion:**

The impairment of executive functions and poor activation of specific neurological circuitry in OCD patients affirms the neurobiological basis of the disorder.

## INTRODUCTION

The impairment of executive functions^1^ and of some specific cognitive functions such as visuo-spatial,^2^ spatial working memory, spatial recognition, motor initiation and execution^3^ along with poor memory functions and organizational capacity,^4^ in patients with the obsessive–compulsive disorder (OCD), despite their average intelligence,^4^,^5^ indicates poor activation of specific neurological circuit responsible for these specific cognitive functions, which implies a neurobiological basis of the disorder. Recent brain imaging and electro-physiological works have consistently shown dysfunction of the fronto-striatal thalamic pathways in OCD subjects.^6^ Moreover, functional MRI studies provide ample evidence of abnormal hyperactivity in orbito-frontal subcortical circuits responsible for the pathogenesis of OCD.^7^ On the other hand, recent studies have revealed the role of the same fronto-striatal circuit in the executive functioning.^3^,^8^ In our previous work as well the executive function has been observed to be impaired in the OCD group.^9^

The close interlinking of similar type of brain substrates and neural circuits in both executive dysfunction as well as in the OCD suggests a possible role of neuropsychological deficit condition in the pathogenesis of OCD. As the executive function is the basic mechanism to regulate the cognitive functions, an executive dysfunction is expected to interfere with the overall information processing system. It is surmised, therefore, that the cognitive style, which determines a mode of processing and organizing cognitive information by disembedding the figure from the ground, may, also be a function of one's neuro-psychological disposition. Since figure-ground visual perception depends on top–down processing of visual stimuli as well,^10^ patients with OCD may well be assumed to encounter difficulty in cognitive differentiation. Although there have been a few attempts to understand the impairment in executive functions related to the OCD group,^9^,^11^ there still exists a paucity of information in literature concerning the effect of neuropsychological disposition on cognitive style and executive functions.

With the objective to better understand the (i) status of executive function and cognitive style in patients with OCD as compared to normal controls, (ii) the neuropsychological disposition and its relationship with executive function and cognitive style in patients with OCD, and, (iii) the relationship of executive functions and cognitive style, a thorough neuropsychological investigation was carried out by employing the Adult Neuropsychological Questionnaire (ANQ), the Wisconsin Card Sorting Test (WCST) and the Embedded Figure Test (EFT).

## METHODS

### Sample

Twenty patients (14 males, 6 females) with OCD diagnosed using the DSM-IV criteria by psychiatrists of the Department of Psychiatry, R.G. Kar Medical College and Hospital, Kolkata, were included in the study. All the selected patients were right-handed, urban individuals, with at least Standard X educational level without having a history of head injury, neurological disease, past psychiatric disorder, alcoholism or any drug abuse. Their age ranged from 18 to 63 years with a mean and standard deviation of 35.65 years and 12.89 years, respectively. The severity of their symptoms was assessed on the Yale-Brown Obsessive Compulsive Scale^14^ and categorized into ‘mild’ (*n*=7), ‘moderate’ (*n*=8) and ‘severe’ (*n*=5) subgroups.

Twenty subjects who matched in all respect with the OCD patients were selected to serve as normal controls. Their age ranged from 18 to 62 years with a mean and standard deviation of 35.55 years and 12.79 years, respectively. These individuals consumed no drugs that are known to affect the functions of the central nervous system. Detailed case history eliciting absence of neurological/neurosurgical problems obtained along with the below cut-off score on Mini Mental Status Examination (MMSE)^12^ and the General Health Questionnaire (GHQ)^13^ prior to their participation was used to screen them for neurological deficit and psychiatric morbidity, respectively.

### Measures

The following measures were used:

- Yale-Brown Obsessive Compulsive Scale (Y-BOCS)^14^
- Wisconsin Card Sorting Test (WCST)^15^
- General Health Questionnaire (GHQ)^13^
- Mini Mental Status Examination (MMSE)^12^
- Adult Neuropsychological Questionnaire (ANQ)^16^
- Embedded Figure Test (EFT)^17^

### Procedure

Following the inclusion and exclusion criteria as mentioned in the ‘sample’, 20 patients with OCD diagnosed by psychiatrists were taken up for the study. First, the Y-BOCS was applied on them and categorized under mild, moderate and severe subgroups. Twenty normal healthy individuals were selected following the inclusion and exclusion criteria and screening through the GHQ and MMSE. All the 40 individuals were administered the WCST to investigate their executive ability. The variables of the WCST considered were: perseverative responses (PR), perseverative errors (PE), non-perseverative errors (NPE), conceptual level response (CLR), number of categories completed (NOCC).

This was followed by administering the ANQ, which provided scores on the neuropsychological status of the following domains: general health (GH), substance abuse (SA), psychiatric problem (PP), general neurological (GN), right hemisphere (RH), left hemisphere (LH), subcortical–cerebeller–spinal (SCS) and sensory or perceptual (S/P).

The EFT was then administered and information was collected on cognitive style of field dependence or independence.

### Statistical treatment

Means (X) and standard deviation (SD) were computed for each of the chosen variables from the WCST, ANQ, EFT. The Mann-Whitney U Test for independent sample was computed for each variable to evaluate the intergroup comparison on all the variables. The difference between the two groups on particular variables was considered as significant only when the probability of occurrence was beyond 0.05 level. The Spearman correlation was computed to find out the relationship between the domains of ANQ and the variables of WCST and EFT.

## RESULTS

**Fig. 1:**
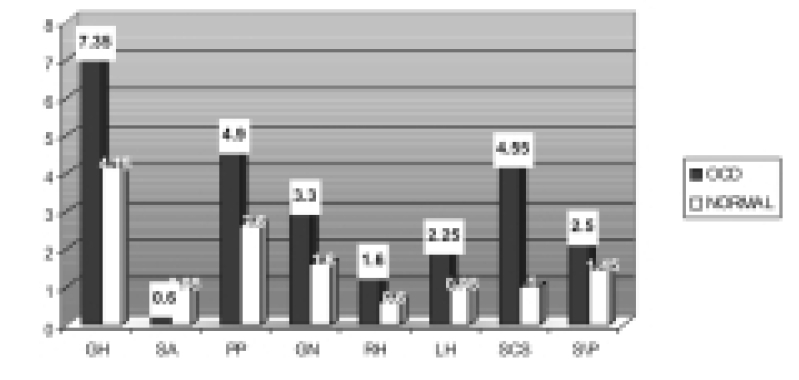
Comparative status of the patients with OCD and normal controls on Adult Neuropsychological Questionnaire

The data obtained on 8 domains of ANQ have been given in Table 1 and graphically represented in Fig. 1. Table 1 shows that the patients with OCD obtained significantly higher scores, denoting a deficit with respect to their neuro-psychological status on all the variables of ANQ except in the domains of SA and S/P. The failure of the domain of SA to distinguish the two groups may be owing to the criteria of exclusion of substance abusers from the study. As expected, the OCD being an anxiety disorder with intact judgement and insight, showed no difference on the domains of S/P function from their normal counterparts.

**Table 1:** Comparative status of the patients with OCD and normal controls on Adult Neuropsychological Questionnaire

	General health	Substance abuse	Psychiatric problem	General neurological	Right hemisphere	left hemisphere	Subcortical-cerebellar-spinal	Sensory perceptual
OCD (Mean)	7.35	0.6	4.9	3.3	1.6	2.25	4.55	2.5
OCD (SD)	3.04	0.66	1.6	1.38	0.97	1.13	2.36	1.75
NORMAL (Mean)	4.15	0.95	2.6	1.6	0.6	0.95	1.0	1.45
NORMAL (SD)	1.79	0.94	1.54	0.88	0.68	0.99	1.03	1.15
U TEST	71	158	63	61	83	79	44	135
ASYM significance	0.000***	0.212	0.000***	0.000***	0.002*	0.001**	0.000***	0.072

* p>0.05;

** p>0.01;

*** p>0.001

**Fig. 2:**
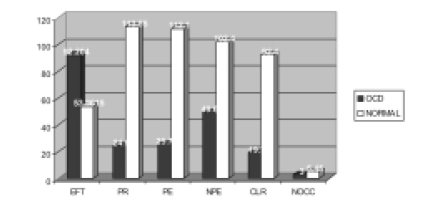
Comparative status of the patients with OCD and normal controls on Embedded Figure Test and Wisconsin Card Sorting Test

The significantly higher mean score on EFT in patients with the OCD in comparison to the normal controls reveals a significantly greater field dependence in them (Table 2, Fig. 2). Low scores on the dimensions of PR, PE, NPE, CLR, and NOCC of WCST in the OCD group reflect executive dysfunction.

**Table 2:** Comparative status of the patients with OCD and normal controls on Embedded Figure Test and Wisconsin Card Sorting Test

	EFT	Perseverative response	Perseverative error	Non-perseverative error	Conceptual level response	Number of category completion
OCD (Mean)	92.2	24.5	25.75	49.65	19.3	3
OCD (SD)	45.08	32.42	35.36	38.27	29.2	2.3
NORMAL (Mean)	53.96	113.55	112.1	102.4	92.4	5.45
NORMAL (SD)	17.85	25.36	30.96	9.95	20.19	0.74
U TEST	79	12.5	18	42.5	22.5	89.5
ASYM significance	0.001***	0.000***	0.000***	0.000***	0.000***	0.002**

* p>0.05;

** p>0.01;

*** p>0.001

Table 3 shows that there exists an inter-correlation between GH and all other variables of ANQ whereas GN and PP were found to be highly correlated with RH, LH, SCS and S/P. An interesting observation is that SCS, SP and PR were closely related to LH deficits and although S/P did not show any significant group difference, its positive correlation to SCS and CLR could have a clinical significance. Considering the general neurological disposition and executive function, it is noted that PP as well as left hemispheric dysfunction is positively related to perseveration (PR and PE) and RH dysfunction is correlated with NPE. Regarding the EFT a positive correlation is obtained with GH and SCS (Table 3).

**Table 3:** Correlation between variables of ANQ and WCST and EFT

Correlated variables		Spearman value	Level of significance
GH	PP	0.589	0.000***
	GN	0.593	0.000**
	LH	0.597	0.000***
	SCS	0.735	0.000***
	S/P	0.666	0.000***
	EFT	0.372	0.018**
PP	GN	0.746	0.000***
	RH	0.408	0.089*
	LH	0.506	0.001***
	SCS	0.544	0.000***
	S/P	0.300	0.009***
	PR	−0.392	0.012**
	PE	−0.491	0.001***
GN	RH	0.317	0.046*
	LH	0.458	0.003**
	SCS	0.669	0.000***
	S/P	0.415	0.008**
RH	NPE	−0.361	0. 022*
LH	SCS	0.615	0.000***
	S/P	0.440	0.005**
	PR	−0.385	0.014**
	PE	−0.372	0.018**
SCS	S/P	0.485	0.002**
	EFT	0.378	0.016**
	CLR	−0.379	0.016**
EFT	NOCC	−0.551	0.000***
			

* p>0.05;

** p>0.01;

*** p>0.001

## DISCUSSION

That the OCD group has impairment in neuropsychological disposition, cognitive style and executive functions is evident from the comparative evaluation of the data obtained from the 2 groups. As a neuropsychological deficit condition is observed in the OCD group, their performance on each of its variables warrants an explanation.

The poor general health condition as reported by the OCD on the ANQ seems to arise from their persistent and uncontrollable thoughts that cause feelings of distress in them, giving rise to anxiety and irritability, which interferes with their everyday functioning. The catabolic conditions initiated by their chronic anxiety state have the possibility to prime their attention bias towards their health status in 50% of the patients in the present study.

The domain of psychiatric problem as assessed on the ANQ reflects the symptoms of OCD, characterized by the presence of recurrent thought and worries. The correlation of PP with RH, LH, SCS and S/P suggests that psychiatric symptoms are associated with the thought which the patients are neither able to suppress nor mould, resulting in the interference with their test performance.

The deficit observed in the general neurological status as felt by the patients in comparison with the normal controls, is in good agreement with the results of other studies, which suggests that OCD symptoms are mediated by multiple brain regions, including anterior cingulate gyrus,^18^ frontal^6^,^19^ and temporal lobes^11^ and basal ganglia.^7^

The greater random error observed through the higher score on the variable of NPE of the WCST in the OCD group in comparison with the normal controls indicates it to be the function of an individual's arousal and attentional system. Accordingly, the significant correlation between neuro-psychological dysfunction of RH and NPE may be attributed to its mediation through RH, owing to the greater reti-culocortical and corticoreticular innervation to the RH than the LH.^20^ The neuropsychological status of the LH in perseveration as has been revealed in the relationship between LH and perseverative response and error, along with the observation of RH dysfunction in NP one, signifies the bilateral involvement in the executive function, and is in agreement with the other research report^2^ based on QEEG which claims that the main pathology of OCD is located in the left hyperfrontality, and that the right hyperfrontality occurs by a compensatory mechanism.

The deficit function of the SCS domain observed from the response of the OCD group corroborates with the findings of the previous researchers who showed impairment in the limbic and subcortical circuitry, specifically in the putamen and caudate nucleus of the basal ganglia,^11^,^21^,^22^ along with a dysfunction of the cerebellum^23^,^24^ in the OCD. The observation of inverse correlation between the SCS of ANQ and conceptual level responses (CLR) of the WCST suggestive of a link between deficit in SCS function and attainment of poor conceptual level response, gets further confirmation from the recent research that reveals the involvement of subcortical centres in cognitive processes, including working memory, rule-based learning, switching attention, visual perception and the planning of future behaviour,^25^ which are the constituents of executive function. This is in agreement with the opinion that cerebellum could be an essential node in the distributed neural circuitry subserving higher-order behaviours.

Regarding cognitive style, an inverse correlation between the score of NOCC and that of EFT suggests an association between field dependence and poor number of category completion, an index of poor cognitive flexibility, indicating less cognitive differentiation with set shifting difficulty confirms that dysexecution^6^ could be detrimental for cognitive differentiation. Perhaps executive function is a necessary condition for the adequate articulation in organizing the ambiguous cognitive data, being independent of the external referents for interpreting it. The finding suggests the possibility that cognitive style being highly correlated with NOCC could be a part of the executive function or, at least, is mediated by the same. The significant correlations of SCS function with both the executive function and the cognitive style suggest that this particular neurological substrate could be intimately associated with both the aforesaid functions.

## CONCLUSION

The performance of the OCD group on the WCST and EFT revealed that they have less cognitive differentiation and impairment in executive function compared to the normal control group. The SCS function as evident on the ANQ is found to be associated with these functions. Moreover, the impaired executive function is also linked with field-dependent cognitive style.

## References

[1] Savage CR, Baer L, Keuthen NJ (1999). Organizational strategies mediate nonverbal memory impairment in obsessive–compulsive disorder. Biol Psychiatry.

[2] Shin YW, Ha TH, Kim SY (2004). Association between EEG alpha power and visuospatial function in obsessive–compulsive disorder. Psychiatry Clin Neurosci.

[3] Purcell R, Maruff P, Kyrios M (1998). Cognitive deficits in obsessive–compulsive disorder on tests of frontal-striatal function. Biol Psychiatry.

[4] Shin MS, Park SJ, Kim MS (2004). Deficits of organizational strategy and visual memory in obsessive–compulsive disorder. Neuropsychology.

[5] Purcell R, Maruff P, Kyrios M (1998). Neuropsychological deficits in obsessive–compulsive disorder: A comparison with unipolar depression, panic disorder, and normal controls. Arch Gen Psychiatry.

[6] Bucci P, Mucci A, Volpe U (2004). Executive hypercontrol in obsessive–compulsive disorder: Electrophysiological and neuropsychological indices. Clin Neurophysiol.

[7] Saxena S, Brody AL, Schwartz JM (1998). Neuroimaging and frontal-subcortical circuitry in obsessive–compulsive disorder. Br J Psychiatry.

[8] Mataix Cols D, Junque C (1999). Neuropsychological functioning in a subclinical obsessive–compulsive sample. Biol Psychiatry.

[9] Mukhopadhyay P, Das S (2003). Problem solving ability in psychiatric patients: A neuropsychological approach. Psychol Res J.

[10] Malaspina D, Simon N, Goetz RR (2004). The reliability and clinical correlates of figure-ground perception in schizophrenia. J Neuropsychiatr Clin Neurosci.

[11] Berthier ML, Kulisevsky J, Gironell A (1996). Obsessive–compulsive disorder associated with brain lesions: Clinical phenomenology, cognitive function, and anatomic correlates. Neurology.

[12] Goldberg DP, Hiller VE (1979). A scaled version of general health questionnaire. Psychol Med.

[13] Strub RL, Black FW (2000). The mental status examination in Neurology.

[14] Goodman WK, Price LH, Rasmussen SA (1989). Yale-brown obsessive–compulsive scale-I: Development, use and reliability. Arch Gen Psychiatry.

[15] Heaton RK, Chelune GJ (1993). Wisconsin card sorting test manual.

[16] Melendez F (1978). Adult Neuropsychological Questionnaire (ANQ).

[17] Witkin HA (1971). A manual of the embedded figure test.

[18] Adler CM, McDonough-Ryan P, Sax KW (2000). MRI of neuronal activation with symptom provocation in unmedicated patients with obsessive–compulsive disorder. J Psychiatr Res.

[19] Amo C, Quesney LF, Ortiz T (2004). Limbic paroxysmal magnetoencephalographic activity in 12 obsessive–compulsive disorder patients: A new diagnostic finding. J Clin Psychiatry.

[20] Lezak MD (1995). Neuropsychological assessment.

[21] Breiter HC, Rauch SL, Kwong KK (1996). Functional magnetic resonance imaging of symptom provocation in obsessive–compulsive disorder. Arch Gen Psychiatry.

[22] Breiter HC, Rauch SL (1996). Functional MRI and the study of OCD: From symptom provocation to cognitive-behavioral probes of cortico-striatal systems and the amygdala. Neuroimage.

[23] Kim JJ, Lee MC, Kim J (2001). Grey matter abnormalities in obsessive–compulsive disorder: Statistical parametric mapping of segmented magnetic resonance images. Br J Psychiatry.

[24] Kang DH, Kwon JS, Kim JJ (2003). Brain glucose metabolic changes associated with neuropsychological improvements after 4 months of treatment in patients with obsessive–compulsive disorder. Acta Psychiatr Scand.

[25] Strick PL, Middleton MA (2001). Cerebellar projections to the prefrontal cortex of the primate. J Neurosci.

